# Neuroprotective Effects of *Albizia lebbeck* (L.) Benth. Leaf Extract against Glutamate-Induced Endoplasmic Reticulum Stress and Apoptosis in Human Microglial Cells

**DOI:** 10.3390/ph16070989

**Published:** 2023-07-10

**Authors:** Onuma Phoraksa, Chanika Chimkerd, Parunya Thiyajai, Kunchit Judprasong, Siriporn Tuntipopipat, Tewin Tencomnao, Somsri Charoenkiatkul, Chawanphat Muangnoi, Monruedee Sukprasansap

**Affiliations:** 1 Doctor of Philosophy Program in Nutrition, Faculty of Medicine Ramathibodi Hospital and Institute of Nutrition, Mahidol University, Bangkok 10400, Thailand; onuma.phoraksa@gmail.com; 2 Center of Analysis for Product Quality (Natural Products Division), Faculty of Pharmacy, Mahidol University, Rajathevi, Bangkok 10400, Thailand; chanika.chm@mahidol.ac.th; 3 Food Chemistry Unit, Institute of Nutrition, Mahidol University, Salaya Campus, Nakhon Pathom 73170, Thailand; parunya.thy@mahidol.ac.th (P.T.); kunchit.jud@mahidol.ac.th (K.J.); somsri.chr@mahidol.ac.th (S.C.); 4 Cell and Animal Model Unit, Institute of Nutrition, Mahidol University, Salaya Campus, Nakhon Pathom 73170, Thailand; siriporn.tun@mahidol.ac.th (S.T.); chawanphat.mua@mahidol.edu (C.M.); 5 Department of Clinical Chemistry, Faculty of Allied Health Sciences, Chulalongkorn University, Bangkok 10330, Thailand; tewin.t@chula.ac.th; 6 Natural Products for Neuroprotection and Anti-Ageing Research Unit, Chulalongkorn University, Bangkok 10330, Thailand; 7 Food Toxicology Unit, Institute of Nutrition, Mahidol University, Salaya Campus, Nakhon Pathom 73170, Thailand

**Keywords:** *Albizia lebbeck* (L.) Benth., apoptosis, ER stress, glutamate, microglial cells, neuroprotection, neurotoxicity

## Abstract

Endoplasmic reticulum (ER) stress caused by excessive glutamate in the central nervous system leads to neurodegeneration. *Albizia lebbeck* (L.) Benth. has been reported to possess neuroprotective properties. We aimed to investigate the effect and mechanism of *A. lebbeck* leaf extracts on glutamate-induced neurotoxicity and apoptosis linked to ER stress using human microglial HMC3 cells. *A. lebbeck* leaves were extracted using hexane (AHE), mixed solvents, and ethanol. Each different extract was evaluated for cytotoxic effects on HMC3 cells, and then non-cytotoxic concentrations of the extracts were pretreated with the cells, followed by glutamate. Our results showed that AHE treatment exhibited the highest protective effect and was thus selected for finding the mechanistic approach. AHE inhibited the specific ER stress proteins (calpain1 and caspase-12). AHE also suppressed the apoptotic proteins (Bax, cytochrome c, cleaved caspase-9, and cleaved caspase-3); however, it also increased the antiapoptotic Bcl-2 protein. Remarkably, AHE increased cellular antioxidant activities (SOD, CAT, and GPx). To support the activation of antioxidant defense and inhibition of apoptosis in our HMC3 cell model, the bioactive phytochemicals within AHE were identified by HPLC analysis. We found that AHE had high levels of carotenoids (α-carotene, β-carotene, and lutein) and flavonoids (quercetin, luteolin, and kaempferol). Our novel findings indicate that AHE can inhibit glutamate-induced neurotoxicity via ER stress and apoptosis signaling pathways by activating cellular antioxidant enzymes in HMC3 cells, suggesting a potential mechanism for neuroprotection. As such, *A. lebbeck* leaf might potentially represent a promising source and novel alternative approach for preventing neurodegenerative diseases.

## 1. Introduction

The prevalence of neurodegenerative diseases has increased constantly in recent decades [1]. Many neurodegenerative diseases, including Alzheimer’s disease (AD) and Parkinson’s disease (PD), are associated with excessive neurotransmitters in the central nervous system (CNS) [2,3]. Glutamate is a major excitatory neurotransmitter in the mammalian CNS, which plays an essential role in nervous system, including neuronal development and transmission. It regulates several functions in the brain, including cognitive, memory, learning, emotional, and endocrine functions [4,5]. In neurons and neuroglia, glutamate binds and activates both ligand-gated ion channels (ionotropic glutamate receptors) and G-protein coupled receptors (metabotropic glutamate receptors) [5]. Glutamate excitotoxicity can cause neuronal and glial cell death in the CNS and is activated by excessive glutamate. Prolonged exposure to glutamate causes excessive activation of glutamate receptors, Ca^2+^ influx, and reactive oxygen species (ROS) accumulation; this can result in mitochondrial dysfunction, endoplasmic reticulum (ER) stress, and cell death, leading to various neurodegenerative disorders [6,7,8]. Microglial cells are one of the main neuroglia types; these cells are macrophages of the CNS and act as the primary innate immune cells in the brain [9]. Numerous reports indicate that prolonged and excessive amounts of glutamate and over-activation of glutamate receptors can lead to microglial neurotoxicity and death, which associate with neurodegeneration [2,8,10,11,12,13].

ER is an important organelle for the synthesis, folding, and transport of proteins as well as the storage of calcium ions [14]. The main pathways of ER-induced apoptosis are the C/EBP homologous protein (CHOP)/GADD153 pathway, the caspase-12 pathway, and the IRE1/ASK1/JNK pathway [15]. An increase of intracellular calcium ions stimulates calpain (a calcium-dependent cysteine protease) to cleave caspase-12. The activated caspase-12 stimulates the apoptotic caspase cascades, such as caspase-9 and caspase-3 [16]. In prolonged or severe ER stress, unfolded protein response (UPR) may lead to apoptosis through PERK-eIF2α-ATF4 signaling, resulting in the induction of transcription factor CHOP and ultimately leading to apoptotic cell death [15,17]. CHOP promotes apoptosis by downregulating the expression of pro-survival proteins (such as B-cell lymphoma 2 (Bcl-2)) and activating the expression of pro-apoptotic proteins (such as Bcl-2-antagonist/killer-1 (Bak) and Bcl-2-associated X protein (Bax)), which also increase the release of cytochrome c from mitochondria [15,18]. ER stress can trigger the activation of several signaling pathways, including the mitogen-activated protein kinase (MAPK) pathway; this pathway relates to cellular proliferation, differentiation, inflammation, and apoptosis. The three main pathways of MAPK are extracellular signal-regulated kinases 1 and 2 (ERK1/2), C-Jun N-terminal kinase/ stress-activated protein kinase (JNK/SAPK), and p38 kinase. The MAPK pathways can be activated by neurotransmitters such as glutamate through glutamate receptors in order to stimulate the expression of target genes [19,20]. JNK and p38 promote apoptosis by activating the pro-apoptotic Bax protein, inhibiting the anti-apoptotic Bcl-2 protein, and increasing the release of cytochrome c and caspase cascade [21]. A previous study revealed that glutamate activates the phosphorylation of JNK in mouse BV2 microglial cells [22]. Moreover, the activation of ERK involves glutamate-induced oxidative toxicity and neuronal cell death [23,24]. Some evidence has been reported that indicates that ER stress promotes ERK activation through PERK-eIF2α signaling, in which the phosphorylated ERK induces apoptosis by activating Bax and caspase cascades signaling [25].

*Albizia lebbeck* (L.) Benth. belongs to the genus Albizia of the *Fabaceae* family. *A. lebbeck* grows in tropical and subtropical regions of deciduous forests in Asian countries, including India, Burma, and Thailand. It is commonly known as Lebbeck, Woman’s-tongue tree, or Ta-kuk. This plant has several active constituents, including flavonoids, carotenoids, saponins, and tannins [26,27]. The leaf extract of *A. lebbeck* exhibits antioxidant [28,29] and anti-inflammatory properties [27,30]. In addition, *A. lebbeck* seed extract has shown a neuroprotective effect in AD and PD rat models [31,32]. Moreover, *A. lebbeck* extracts increased the levels and activities of antioxidant enzymes such as superoxide dismutase (SOD), catalase (CAT), glutathione peroxidase (GPx), and glutathione-S transferase (GST) [31,33,34]. However, there is no study using *A. lebbeck* leaf on human microglial cells. Therefore, this is the first study to investigate the protective effects and underlying mechanisms of *A. lebbeck* leaf extracts on glutamate-induced neurotoxicity in human microglial HMC3 cells through ER stress and apoptosis pathways.

## 2. Results

### 2.1. The Cytotoxicity of Different Solvent Extracts from A. lebbeck Leaf on Cell Viability of HMC3 Cells

**Figure 1:**
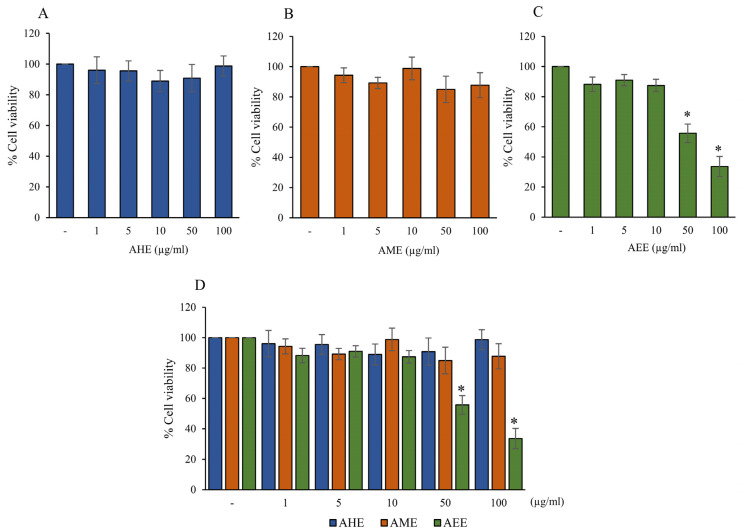
Effect of different solvents extracts from *A. lebbeck* leaf on cell viability of HMC3 cells. Cells were treated with various concentrations of (**A**) AHE, (**B**) AME, and (**C**) AEE for 24 h and cell viability was determined by MTT assay. (**D**) The bar graph compares the % cell viability of three solvent extracts. The basal media containing 0.2% DMSO was used as the negative control. Data are expressed as mean ± SD (*n* = 3); * *p* < 0.05 compared to the control group. (AHE, *A. lebbeck* leaf hexane extract; AME, *A. lebbeck* leaf mixed solvent extract; AEE, *A. lebbeck* leaf ethanol extract).

To investigate the effects of different solvent extracts from *A. lebbeck* leaf using various polarities of organic solvents on protection against glutamate toxicity in human microglial HMC3 cells, we initially evaluated the non-cytotoxic or cytotoxic concentrations of the extracts. We determined this by MTT assay. In this study, *A. lebbeck* leaves were extracted with three different polarities of organic solvents, including hexane (non-polarity), mixed solvents (moderate polarity), and 95% ethanol (high polarity). The HMC3 cells were treated with three different *A. lebbeck* leaf extracts (AHE, AME, and AEE) at various concentrations (0–100 µg/mL) for 24 h. Our results showed that treatments of the cells with AHE or AME at 1–100 µg/mL were non-toxic to cells when compared with the control cells, as shown in Figure 1A,B. The percentages of cell viability were more than 80%. In contrast, AEE showed toxicity to cells at 50–100 µg/mL. This significantly decreased cell viability by more than 40% when compared to the control cells (Figure 1C). Based on this data, the comparison of three solvent extracts demonstrated that AEE was the most cytotoxic to cells. The highest concentration (10 µg/mL) of all the AHE, AME, and AEE extracts did not show any significant toxic effect in HCM3 cells (Figure 1D). Thus, this concentration was selected to examine the protective effects in subsequent experiments. 

### 2.2. The Protective Effect of A. lebbeck Leaf Extracts on Glutamate-Induced Toxicity in HMC3 Cells

**Figure 2:**
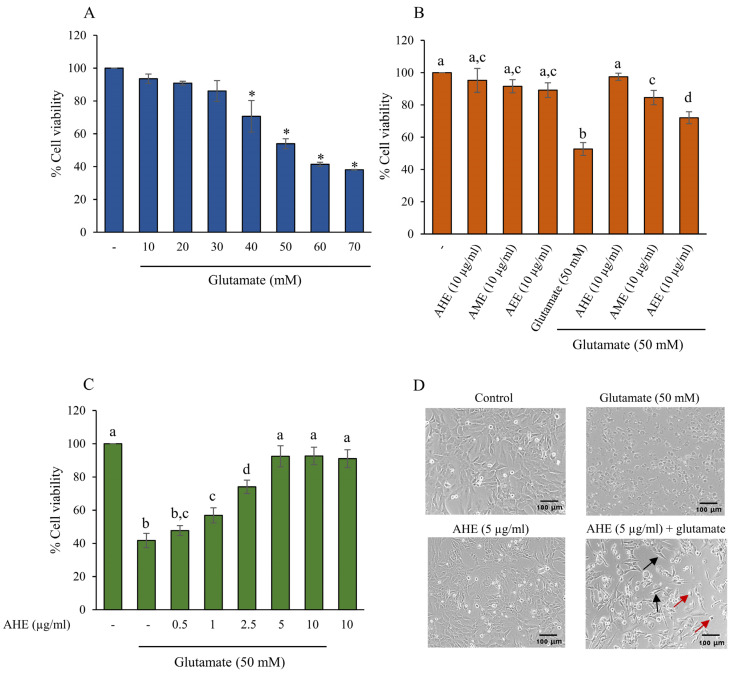
Effect of glutamate on cell viability and three different extracts on glutamate-induced toxicity in HMC3 cells. The cell viability was evaluated by MTT assay. (**A**) Cells were treated with various concentrations of glutamate for 24 h. (**B**) The cells were pre-treated with 10 µg/mL of AHE, AME, or AEE for 24 h, followed by glutamate (50 mM) for 24 h. (**C**) Cells were pre-treated with various concentrations of AHE for 24 h, followed by glutamate (50 mM) for 24 h. (**D**) After the specific treatment, morphology of HMC3 cells was observed using a microscope with 10x magnification (scale bar is 100 µm). The black arrow indicates the elongated cells. The red arrow represents the round cells. Data are expressed as mean ± SD (*n* = 3); * *p* < 0.05 compared to the control group. Different letters (a–d) indicate significant differences among groups with *p* < 0.05. (AHE, *A. lebbeck* leaf hexane extract; AME, *A. lebbeck* leaf mixed solvent extract; AEE, *A. lebbeck* leaf ethanol extract).

To assess the protective effect of the extracts on glutamate-induced cytotoxicity in HMC3 cells, we first evaluated the level of glutamate toxicity that caused HCM3 cell injury and death. The HMC3 cells were incubated with glutamate at various concentrations (0–70 mM) for 24 h, and the cell viability was measured by MTT assay. The results showed that glutamate concentration at 40–70 mM significantly decreased cell viability in a dose-dependent manner (Figure 2A). Treating cells with 50 mM glutamate noticeably reduced cell viability by around 50% when compared with the control cells, as shown in Figure 2A. Therefore, this study used glutamate concentration at 50 mM in order to induce and indicate the neurotoxicity of our HMC3 microglial cell model. 

Next, we compared the protective effects of three different polarity extracts from *A. lebbeck* leaf against glutamate-induced toxicity in HMC3 cells. The cells were pretreated with 10 µg/mL AHE, AME, or AEE for 24 h prior to incubation with 50 mM glutamate for 24 h. Then, cell viability was assessed by MTT assay. As shown in Figure 2B, the glutamate treatment group significantly suppressed cell viability by about 50%. Pretreatment of cells with all three extracts significantly increased cell viability when compared to the glutamate group, with the AHE treatment exhibiting the maximum protective effect (Figure 2B). AHE was therefore selected for the following experiments. HMC3 cells were then pre-treated with AHE at 0.5–10 µg/mL for 24 h, followed by 50 mM glutamate. We found that pretreatment of cells with AHE (1–10 µg/mL) significantly inhibited glutamate-induced toxicity when compared with the glutamate-alone group (Figure 2C). Since there was no significant difference at concentrations of 5 and 10 µg/mL AHE, AHE at 5 µg/mL was chosen as the optimum dose for subsequent investigations of the mechanistic approach. Additionally, we also observed cell morphology under a light microscope. The glutamate-treated cells appeared round with abnormal shape and structure, whereas cells pre-treated with AHE maintained the cell morphology (Figure 2D). Taken together, these results suggest that pretreatment of cells with AHE potentially protects against glutamate-induced cytotoxicity in our HMC3 cell model.

### 2.3. AHE Suppresses Glutamate-Induced ER Stress in HMC3 Cells

**Figure 3:**
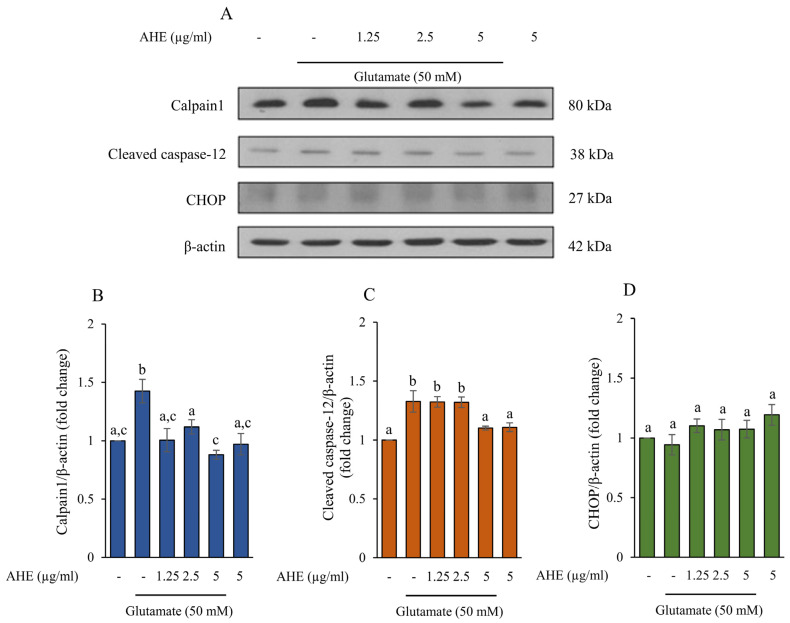
Effect of AHE treatment on glutamate-induced protein expression of ER stress markers in HMC3 cells. (**A**) Cells were pre-treated with various concentrations of AHE for 24 h and then incubated with glutamate (50 mM) for 30 min. (**A**) The protein expressions of calpain1, caspase-12, and CHOP were measured by Western blot analysis. The quantitative protein levels of (**B**) calpain1, (**C**) cleaved caspase-12, and (**D**) CHOP were quantified by densitometry and normalized with ꞵ-actin. Data are expressed as mean ± SD (*n* = 3). Different letters (a–c) indicate significant differences among groups with *p* < 0.05. (AHE, *A. lebbeck* leaf hexane extract).

The high concentration and accumulation of glutamate are involved in ER stress in the CNS, leading to apoptotic cell death. This event can stimulate the specific ER stress proteins, including the calcium ion-mediated calpain/caspase-12 pathway or the CHOP pathway [35,36,37,38]. To examine the effect of AHE on ER stress-induced apoptosis caused by glutamate, we evaluated protein expression of the markers of ER stress, including calpain1, cleaved caspase-12, and CHOP, using Western blot analysis. HMC3 cells were incubated with AHE at concentrations of 0–5 µg/mL for 24 h and then exposed to 50 mM glutamate for 30 min. As shown in Figure 3A–C, the protein expressions of calpain1 and cleaved caspase-12 significantly increased in the glutamate-treated group when compared with the control group. However, the glutamate treatment did not affect the level of CHOP expression when compared with the control group (Figure 3A,D). Remarkably, the pretreatment of cells with AHE could inhibit the expressions of calpain1 and cleaved caspase-12 (Figure 3A–C), with the highest inhibitory effect at a concentration of 5 µg/mL. The original blots were shown in the Supplementary Material (Figure S1). Our results indicate that pretreated cells with AHE attenuates glutamate-induced ER stress by suppressing the calpain1 and cleaved caspase-12 protein expressions in HMC3 cells.

### 2.4. Effect of AHE on MAPKs Activation in HMC3 Cells

**Figure 4:**
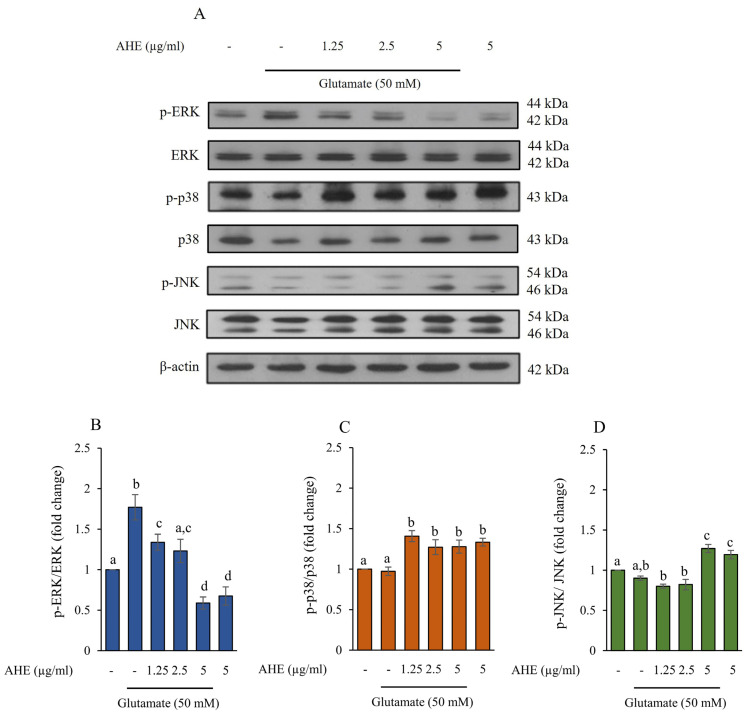
Effect of AHE treatment on glutamate-induced MAPKs protein expression in HMC3 cells. (**A**) Cells were pre-treated with various concentrations of AHE for 24 h and then exposed to glutamate (50 mM) for 30 min. (**A**) The protein expressions of ERK, p38, and JNK were measured using Western blot analysis. The quantitative protein levels of (**B**) phosphorylated ERK/ERK, (**C**) phosphorylated p38/p38, and (**D**) phosphorylated JNK/JNK were quantified by densitometry and normalized with ꞵ-actin. Data are expressed as mean ± SD (*n* = 3). Different letters (a–d) indicate significant differences among groups with *p* < 0.05. (AHE, *A. lebbeck* leaf hexane extract).

In the nervous system, elevated glutamate can provoke the MAPKs (ERK, p38, and JNK) signaling pathway. This pathway is involved in cellular proliferation, differentiation, inflammation, and apoptosis [20,39,40]. To assess the effect of AHE on glutamate-induced MAPKs activation in HMC3 cells, we investigated whether AHE might alter the expression of these proteins. The cells were pre-treated with AHE (0–5 µg/mL) for 24 h prior to co-incubation with 50 mM glutamate for 30 min. Then, the protein expression levels of total ERK, active form of ERK (phosphorylated ERK; p-ERK), total p38, active form of p38 (phosphorylated p38; p-p38), total JNK, and active form of JNK (phosphorylated JNK; p-JNK) were examined using Western blot analysis. As illustrated in Figure 4A–D, the results showed that glutamate treatment alone dramatically increased p-ERK expression but the expressions of p-p38 and p-JNK were not significantly altered when compared with the non-treated control cells. The pretreatment of cells with AHE significantly decreased p-ERK in a concentration-dependent manner (Figure 4A,B), whereas AHE (1.25–5 µg/mL) significantly increased the expression of p-p38 (Figure 4A,C). AHE at only 5 µg/mL significantly raised the expression of p-JNK (Figure 4A,D) when compared with glutamate treatment. The original blots were shown in the Supplementary Material (Figure S2). These results suggest that the pretreatment of AHE could reduce the MAPKs activation by suppressing ERK phosphorylation.

### 2.5. AHE Inhibits Glutamate-Induced Apoptosis and Caspase Activities in HMC3 Cells

**Figure 5:**
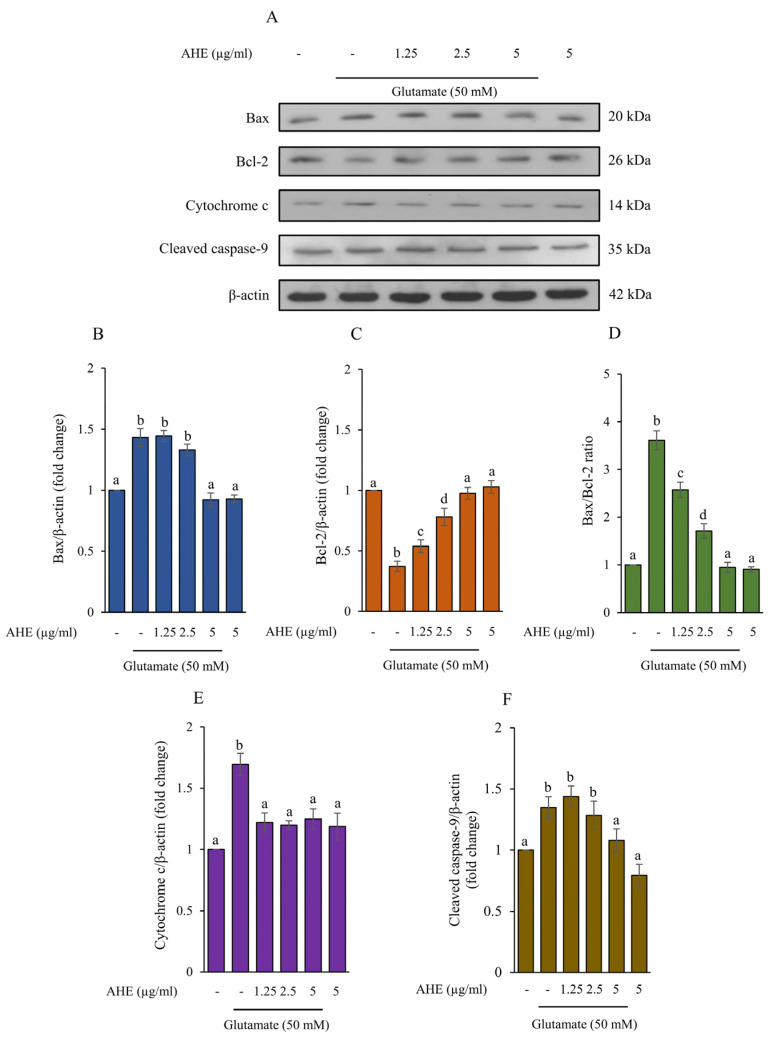
Effect of AHE treatment on glutamate-induced expression of apoptosis-related proteins in HMC3 cells. Cells were pre-treated with various concentrations of AHE for 24 h, followed by glutamate (50 mM) for 30 min. (**A**) The expressions of Bax, Bcl-2, cytochrome c, and cleaved caspase-9 were measured using Western blot analysis. The quantitative protein levels of (**B**) Bax, (**C**) Bcl-2, (**D**) Bax/Bcl-2, (**E**) cytochrome c, and (**F**) cleaved caspase-9 were quantified by densitometry and normalized with ꞵ-actin. Data are expressed as mean ± SD (*n* = 3). Different letters (a–d) indicate significant differences among groups with *p* < 0.05. (AHE, *A. lebbeck* leaf hexane extract).

**Figure 6:**
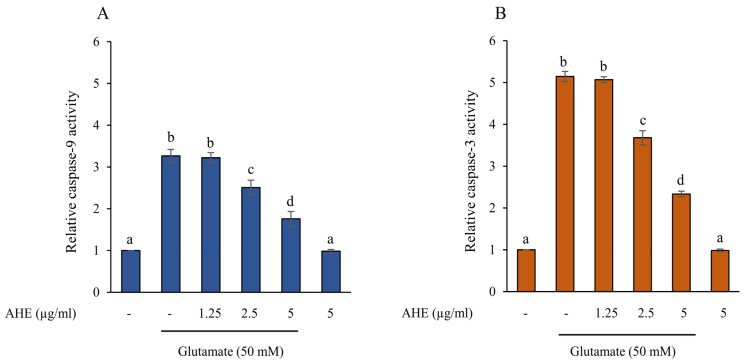
Effect of AHE treatment on glutamate-induced activities of caspase-9 and caspase-3 in HMC3 cells. Cells were pre-treated with various concentrations of AHE for 24 h and then incubated with glutamate (50 mM) for 30 min. The activities of (**A**) caspase-9 and (**B**) caspase-3 were measured using an assay kit. The results are presented as the relative activity. Data are expressed as mean ± SD (*n* = 3). Different letters (a–d) indicate significant differences among groups with *p* < 0.05. (AHE, *A. lebbeck* leaf hexane extract).

Excessive glutamate can cause neuronal cell death by increasing apoptotic protein levels, such as Bax, cytochrome c, caspase-9, and caspase-3, while decreasing anti-apoptotic protein levels, including Bcl-2 [35,41,42,43]. To further illustrate the effect of AHE on glutamate-induced apoptosis, the protein expressions of apoptotic markers, including Bax, Bcl-2, cytochrome c, and cleaved caspase-9, were determined using Western blot analysis. The HMC3 cells were pre-treated with various concentrations of AHE (1.25–5 µg/mL) for 24 h and exposed to glutamate for 30 min. We found that the cells treated with glutamate alone significantly increased the protein expressions of Bax, cytochrome c, and cleaved caspase-9, while notably reducing the expression of Bcl-2 when compared with the non-treated control cells (Figure 5). Interestingly, pretreatment of cells with AHE significantly inhibited the expression of Bax, cytochrome c, and cleaved caspase-9 (Figure 5A,B,E,F) and increased the expression of Bcl-2 in a concentration-dependent manner when compared with the glutamate treatment (Figure 5A,C). Additionally, the ratio of Bax/Bcl-2 was shown to be associated with apoptosis in our HMC3 cell model, as presented in Figure 5D. The original blots were shown in the Supplementary Material (Figure S3). Furthermore, we also evaluated the activities of caspase-9 and caspase-3 using assay kits. Results showed that cells pre-treated with AHE (1.25–5 µg/mL) significantly reduced caspase-9 and caspase-3 activities in a dose-dependent manner when compared with glutamate treatment (Figure 6A,B). Taken together, our results demonstrate that AHE inhibits the glutamate-induced apoptosis signaling pathway by stimulating the anti-apoptotic Bcl-2 protein and repressing apoptotic Bax, cytochrome c, caspase-9, and caspase-3 proteins, indicating the ability of AHE to protect against glutamate in HMC3 cells.

### 2.6. AHE Promotes Antioxidant Enzymes Activity in HMC3 Cells

**Figure 7:**
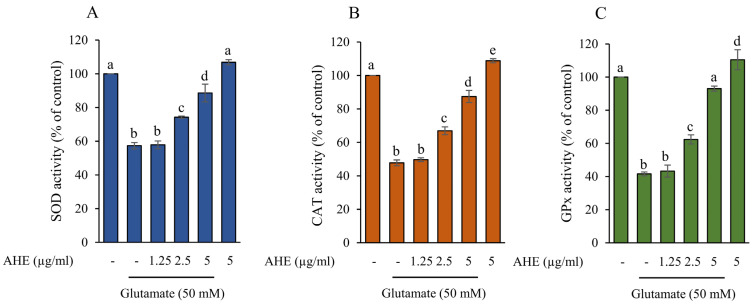
Effect of AHE treatment on antioxidant enzyme activity in HMC3 cells. Cells were pre-treated with various concentrations of AHE for 24 h, followed by glutamate (50 mM) for 30 min. The activities of (**A**) SOD, (**B**) CAT, and (**C**) GPx were measured using an assay kit. Data are expressed as mean ± SD (*n* = 3). Different letters (a–e) indicate significant differences among groups with *p* < 0.05. (AHE, *A. lebbeck* leaf hexane extract).

An excessive glutamate level relates to a decrease in the antioxidant system, leading to microglial cell death [35,44,45]. The primary antioxidant enzymes in the nervous system include SOD, CAT, and GPx; these prevent cellular oxidative damage [46,47]. To further confirm the antioxidant defense of AHE against glutamate-induced neurotoxicity in our HMC3 cell model, cells were pre-incubated with AHE at different concentrations (1.25–5 µg/mL), followed by 50 mM glutamate for 30 min. The activities of antioxidant enzymes, such as SOD1, CAT, and GPx, were measured using assay kits. Our results clearly demonstrated that pretreatment of cells with AHE significantly upregulated SOD, CAT, and GPx activities in a concentration-dependent manner when compared with the glutamate treatment, while glutamate alone showed a considerable decrease when compared to non-treated control cells (Figure 7A–C). Interestingly, the highest dose of AHE treatment alone, at 5 µg/mL, significantly enhanced all three antioxidant enzyme activities. These results indicate that AHE can promote cellular antioxidant enzymes and prevent glutamate-induced neurotoxicity in HMC3 cells.

### 2.7. Carotenoids and Flavonoids in AHE

**Figure 8:**
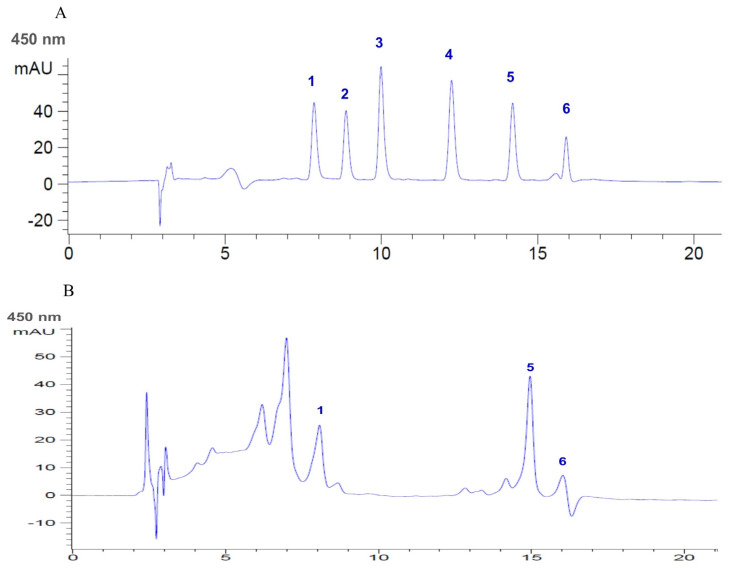
HPLC chromatograms of (**A**) carotenoid standards and (**B**) *A. lebbeck* leaf hexane extract (AHE). (1. lutein; 2. zeaxanthin; 3. α-crytoxanthin; 4. β-crytoxanthin; 5. α-carotene; 6. β-carotene).

**Figure 9:**
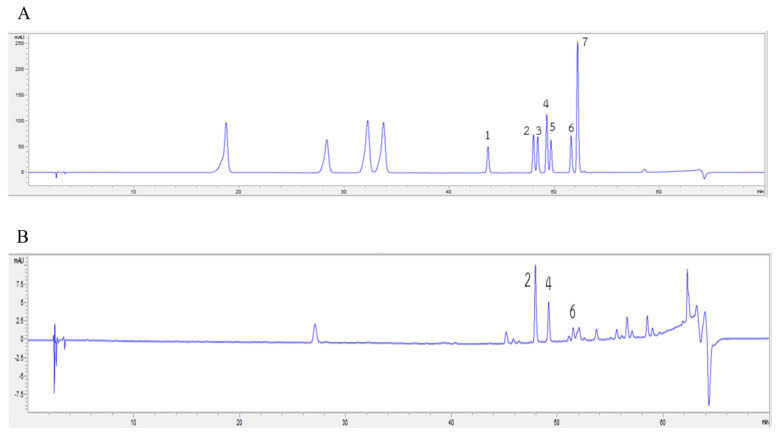
HPLC chromatograms of (**A**) flavonoid standards and (**B**) *A. lebbeck* leaf hexane extract (AHE). (1 = myricetin; 2 = quercetin; 3 = naringenin; 4 = luteolin; 5 = hesperitin; 6 = kaempferol; 7 = apigenin).

Our cell-based and mechanistic investigations found that the AHE treatment showed the maximal protective effects against glutamate-induced neurotoxicity and cell death, as shown in the above data. Therefore, to determine the phytochemical components within AHE that support the activation of antioxidant defense and inhibition of apoptosis in our HMC3 cell model, the phytochemical constituents within AHE were analyzed using the HPLC technique. Results showed that AHE had high carotenoids and flavonoids content. The levels of each carotenoid and flavonoid found in AHE were identified as presented in Table 1. The main carotenoids components found in AHE included α-carotene (4706.57 ± 37.05 µg/100 g FW), lutein (4137.28 ± 162.20 µg/100 g FW), and β-carotene (819.32 ± 24.85 µg/100 g FW); zeaxanthin and β-cryptoxanthin were undetected. Total carotenoid content was equal to 9663.17 ± 224.10 µg/100 g FW. In addition, flavonoid components were also detected, namely quercetin (989.42 ± 53.40 µg/g FW), luteolin (254.67 ± 9.83 µg/g FW), and kaempferol (103.41 ± 9.05 µg/g FW); apigenin, hesperitin, myricetin, and narigenin were not detected. The total flavonoid content amounted to 1347.50 ± 72.28 µg/g FW. Additionally, we produced the HPLC chromatograms of carotenoid and flavonoid standards and AHE to confirm the main components of carotenoids were α-carotene, β-carotene, and lutein (Figure 8); flavonoids were quercetin, luteolin, and kaempferol (Figure 9). These data suggest that AHE shows high phytochemical components in both carotenoid and flavonoid groups which act as potent antioxidant and neuroprotectant, leading to the stimulation of antioxidant enzymes activities and inhibition of apoptotic cell death in human microglial HMC3 cells.

## 3. Discussion 

Microglial cells are one of the neuroglia that act as primary innate immune cells or macrophages for immune defense, injury repair, homeostasis maintenance, and neuronal protection in the CNS, where they are associated with the pathogenesis of various neurodegenerative diseases [48,49,50]. The progressive loss of microglia in the CNS characterizes neurodegenerative disorders, including dementia or AD [51]. The main molecular features of these diseases involve excitotoxicity, ER and mitochondrial stress dysfunctions, neuroinflammation, and oxidative stress, resulting in neurotoxicity and cell death [6,7,8,52]. Excitotoxicity refers to neuronal or microglial cell injury and death due to excessive or prolonged exposure to excitatory amino acids, especially glutamate [3,5,6,7,35]. Glutamate is a key excitatory neurotransmitter in the mammalian CNS that performs an essential role in neuronal development and transmission [4,5]. It binds and activates both ligand-gated ion channels and G-protein coupled receptors. Microglia express several glutamate receptors; thus, prolonged exposure to glutamate leads to excessive activation of the glutamate receptors. This can cause high intracellular calcium ion levels, oxidative stress, mitochondrial dysfunction, and ER stress, contributing to neuronal or microglia excitotoxicity and neurodegenerative processes in AD [2,3,5,7,35,53]. The high concentration of glutamate stimulates intracellular calcium ion influx; this results in calpain activation and promotes caspase-12. Caspase-12 plays a specific role in ER stress-induced apoptosis through the activation of downstream caspases, such as caspase-9 and caspase-7. Eventually, caspase-3 is activated to promote apoptosis [20,35,53,54]. CHOP is another specific protein involved in ER stress-induced apoptosis. CHOP activates the pro-apoptotic proteins and inhibits the anti-apoptotic protein, namely Bcl-2, resulting in apoptosis of cells [54]. Therefore, the suppression of glutamate-induced neurotoxicity related to ER stress and apoptosis may provide a good strategy for a protective approach in neurodegenerative diseases.

The present study demonstrated that glutamate treatment could cause toxicity and ER stress, resulting in apoptosis in our human microglia HMC3 cell model. Consequently, we investigated the protein expressions of specific ER stress, including calpain1, caspase-12, and CHOP exposed to glutamate. Next, the apoptosis markers, such as Bcl-2, Bax, cytochrome c, caspase-9, and caspase-3, were examined (Figure 5). The glutamate treatment upregulated the protein expressions of calpain1 and cleaved caspase-12 but did not increase the expression of CHOP in our cell model (Figure 3). A previous study indicated that ER stress-induced apoptosis occurred independently of CHOP activation [54]. In addition, the results also revealed that the protein expressions of apoptotic markers, including Bax, cytochrome c, and cleaved caspase-9, notably increased in the glutamate treatment group, while the protein expression of anti-apoptotic Bcl-2 dramatically decreased (Figure 5). Moreover, glutamate treatment also stimulated the activities of caspase-9 and caspase-3, which led to HMC3 cell death (Figure 6). Several studies revealed that glutamate induced neuronal death by stimulation of apoptosis markers expression, including Bax, cytochrome c, cleaved caspase-9, and cleaved caspase-3 [41,55,56]. These findings confirmed that glutamate-induced neurotoxicity and ER stress are an important mechanisms linking to apoptosis in these HMC3 cells. 

For a neuroprotective approach, our results indicated that pretreatment of HMC3 cells with AHE obviously inhibited the expressions and activities of these ER stress markers and apoptotic proteins and significantly increased the expression of anti-apoptotic Bcl-2 protein in response to glutamate treatment (Figure 3, Figure 5 and Figure 6). Additionally, numerous works reported that prolonged ER stress induced the activation of MAPK pathways and promoted apoptosis in the nervous system [15,19,20]. MAPK pathways play an important role in cellular proliferation, differentiation, inflammation, and apoptosis. This pathway can be activated by neurotransmitters such as glutamate through glutamate receptors to stimulate the expression of targeted genes/proteins [20,40]. We found that glutamate could induce toxicity via activation of ERK phosphorylation in HMC3 cells. Interestingly, the pretreatment of cells with AHE protected against toxicity by reducing ERK phosphorylation (Figure 4A,B). Several studies demonstrated glutamate-induced toxicity through activation of ERK, which is associated with cell death in many neuronal death models, including microglial cells [37,41,57,58]. 

Furthermore, our investigation showed the inhibitory effect of AHE on glutamate-induced ER stress and apoptosis linked to cellular antioxidant enzymes defense in HMC3 cells. We found that pretreatment of cells with AHE clearly promoted the activity of antioxidant enzymes such as SOD, CAT, and GPx, whereas these enzyme activities significantly decreased in the glutamate group (Figure 7). Some reports revealed glutamate-induced cytotoxicity through the reduction of antioxidant enzyme activity [35,44,45]. Previous studies revealed that the *A. lebbeck* extract exhibited antioxidant activities [28,29,59] and neuroprotective effects in the AD and PD rat models [31,32]. Moreover, some evidence showed that *A. lebbeck* extract could increase levels and activity of antioxidant enzymes such as SOD, CAT, and GPx [31,33]. Since our results on the protective effect of *A. lebbeck* leaf extract indicated that AHE showed the highest protective effect against glutamate-induced cell death in HMC3 cells, we also identified the major bioactive compounds within AHE by HPLC analysis. The results revealed that AHE contained significant amounts of carotenoids (α-carotene, lutein, and β-carotene) and flavonoids (quercetin, luteolin, and kaempferol) (Table 1). Many studies indicate that these compounds can cross the blood-brain barrier and exhibit neuroprotective activity. They showed antioxidant and anti-apoptotic activities via induction of the antioxidant pathway and reduction of the apoptotic pathway [60,61,62]. Flavonoids and carotenoids have displayed protective effects against oxidative stress-induced toxicity in neuronal cells, while carotenoids attenuate glutamate-induced toxicity by inhibition of oxidative stress, apoptosis, and upregulation of antioxidant enzymes [63]. Moreover, quercetin inhibits glutamate-induced toxicity by reduction of MAPK pathways phosphorylation, upregulation of antioxidant systems, and downregulation of pro-apoptotic proteins such as Bid, Bax, and cytochrome c [64,65]. Quercetin and luteolin could reduce ER stress-induced apoptosis by inhibiting CHOP, Bax, and cleaved caspase-3 and increasing Bcl-2 [14]. In addition, previous studies reported that *A. lebbeck* seed extract showed neuroprotective effects in AD and PD rat models [31,32]. Kaempferol is one of the compounds of *A. lebbeck* that relates to therapeutic function in PD [32]. Some studies indicated that the essential oils from the *A. lebbeck* leaf have anti-nociceptive and anti-inflammatory activities [66]. These effects may be caused by various bioactive compounds within *A. lebbeck* leaves. Interestingly, our results revealed that AHE contained bioactive carotenoids and some flavonoid compounds with various non-polar components indicating neuroprotective effects. *A. lebbeck* leaves contain several phytochemicals [26,27] with broad pharmacological activities [27,28,29,30,31,32]. As such, other bioactive components or phytochemicals within AHE may provide protective and synergistic effects to suppress glutamate-induced toxicity and apoptosis in human microglial HMC3 cells. However, the effect of AHE and its major components on normal cell lines and in vivo should be studied, and other underlying mechanisms should be clarified in further investigations. 

Our data demonstrate that AHE consists of several phytochemical components of both carotenoids and flavonoids; these act as potent antioxidants and neuroprotectants. Their synergistic effects protect against glutamate-induced toxicity in our cell model. Collectively, our findings suggest that AHE treatment could prevent glutamate-induced ER stress and cell death by upregulating anti-apoptotic proteins and downregulating pro-apoptotic proteins related to the antioxidant defense activation in human microglial HMC3 cells.

## 4. Materials and Methods

### 4.1. Chemicals and Reagents

Dulbecco’s modified Eagle’s medium (DMEM), minimum essential media (MEM), sodium pyruvate, and trypsin-EDTA were purchased from Gibco (Grand Island, NY, USA). Fetal bovine serum (FBS) was purchased from Merk (Darmstadt, Germany). Penicillin and streptomycin solution were purchased from Caisson Labs (Smithfield, UT, USA). Non-essential amino acids were purchased from Cambrex Bio Science (Walkersville, MD, USA). L-glutamic acid monosodium salt, dimethyl sulfoxide (DMSO), and other chemicals were purchased from Sigma Chemical Co. (St. Louis, MO, USA). The primary antibodies were purchased from Cell Signaling Technology (Danvers, MA, USA) and Abcam (Cambridge, UK). The secondary antibodies were purchased from Cell Signaling Technology (Danvers, MA, USA). All standard compounds of carotenoids and flavonoids (purity ≥ 99%) were purchased from Sigma-Aldrich Co. (St. Louis, MO, USA).

### 4.2. Sample Collection and Preparation

Young leaves of *A. lebbeck* were collected during February and March from the conservation area of the Electricity Generating Authority of Thailand, Srinakarind Dam, Kanchanaburi province, initiated by Plant Genetic Conservation Project under the Royal Initiation of Her Royal Highness Princess Maha Chakri Sirindhorn (RSPG). This plant was identified by and the scientific name confirmed by Assistant Professor Dr. Thaya Jenjittikul, Department of Plant Science, Faculty of Science, Mahidol University, Thailand. A voucher specimen (Code No: 9429) was deposited at Suan Luang Rama IX Herbarium, Bangkok, Thailand. After collection, the leaves were washed with tap water, rinsed with deionized water, and air-dried. The edible young leaves were weighed and cooked in boiling water (ratio 1:10 (*w*/*v*)) for 2 min, based on the method commonly used for consumption by local Thai people [27,67]. After being air-dried, the cooked samples were weighed and then homogenized using an electric blender and freeze dried. Finally, the lyophilized sample was ground to a fine powder, packed under a vacuum in laminated aluminum foil bags, and stored at −20 °C until further use.

### 4.3. Sample Extraction

The lyophilized samples were extracted with different polarities of organic solvents, namely hexane, mixed solvents (hexane: acetone: ethanol at a ratio of 2:1:1 (*v*/*v*/*v*), and 95% ethanol at a solvent ratio of 1:15 (*w*/*v*). Briefly, each sample was mixed with different solvents using a vortex mixer and soaked in darkness at room temperature overnight (16–18 h). After that, they were sonicated in an ultrasonic bath (DAIHAN Scientific, Gangwon-do, Korea) at 25 °C for 10 min, and centrifuged at 4600 rpm (Hettich, Rotina 38R centrifuges, Tuttlingen, Germany) for 10 min. The supernatant was transferred into a flat bottom flask while each sample sediment was further extracted twice with three different solvents. The extracted solvent was evaporated with a vacuum rotary evaporator (Buchi Rotavapor R-114, Flawil, Switzerland) at 40 °C until dry. The yield of the young leaves of *A. lebbeck* from hexane extract (AHE), mixed solvent extract (AME), and ethanol extract (AEE) were 2.10%, 5.70%, and 8.55%, respectively. Each crude extract was kept in darkness and stored at −20 °C.

### 4.4. Cell Culture

Human microglial clone 3 or HMC3 cell line (ATCC^®^ CRL-3304 ^TM^) was purchased from the American Type Culture Collection (ATCC, Rockville, MD, USA). The cells were cultured in a T-75 flask (75 cm^2^ cell culture flask) and maintained with a complete medium at 37 °C in a humidified incubator (Thermo Scientific, Marietta, OH, USA) with 5% CO_2_. The complete medium comprised of 10% (*v*/*v*) heat inactivated FBS, 100 U/mL penicillin, 100 μg/mL streptomycin (antibiotic), 1% (*v*/*v*) non-essential amino acids, and 1 mM sodium pyruvate in basal media (MEM). The adhered cells were grown to around 80–90% confluence before being used for the experiments. Cells used were between passage numbers 2–12.

### 4.5. Cytotoxicity of the Extracts and Glutamate in HMC3 Cells

The cytotoxicity of *A. lebbeck* extracts and glutamate of HMC3 cell line was evaluated using 3-(4,5-dimethylthiazol-2-yl)-2,5-diphenyltetrazolium bromide (MTT) assay. Each of the three extracts were dissolved in DMSO. HMC3 cells were seeded in 48-well plates at a density of 7.5 × 10^4^ cells/well for 24 h. Then, cells were treated with different concentrations of *A. lebbeck* extracts (1–100 µg/mL) in basal media or L-glutamate (10–70 mM) for 24 h. The basal media containing 0.2% DMSO and the basal media were used as the negative control for cytotoxic studies of *A. lebbeck* extracts and L-glutamate, respectively. Then, the culture supernatants were discarded and the MTT reagent (5 mg/mL in PBS) was added to each well. Cells were incubated at 37 °C, 5% CO_2_ for 3 h. Next, the cell supernatants were discarded, followed by DMSO for solubilizing the formazan crystals. Finally, absorbance at 540 nm was measured by a microplate reader (Bio Tek Instruments, Highland, Winooski, VT, USA). Results were presented as the percentage of cell viability compared to the control cells group.

### 4.6. Western Blot Analysis

The HMC3 cells were seeded in 6-well plates at a density of 1.5 × 10^5^ cells/well for 24 h. Then, cells were pre-treated with the selected extract for 24 h followed by glutamate. Afterwards, the treated cells were washed with cold PBS and lysed on ice with cell lysis buffer containing protease and phosphatase inhibitor (Cell signaling, Danvers, MA, USA). The cell lysates were collected by centrifugation at 13,500 rpm at 4 °C for 10 min. The protein concentration was measured by the bicinchoninic acid (BCA) method and bovine serum albumin (BSA) used as a standard. The amounts of protein samples (40 µg) were separated by 10% SDS-polyacrylamide gel electrophoresis (SDS-PAGE) and transferred onto nitrocellulose membranes (Sigma Aldrich, Dorset, UK). The membranes were blocked with blocking buffer and washed with Tris-buffered saline containing 0.1% Tween-20 (TBST). Then, the membranes were incubated at 4 °C overnight with the primary antibodies. These antibodies, namely calpain1 (1:1000), CHOP (1:1000), cleaved caspase-9 (1:1000), cytochrome c (1:1000), Bax (1:1000), Bcl-2 (1:1000), p-ERK (1:1000), ERK (1:1000), p-p38 (1:1000), p38 (1:1000), p-JNK (1:1000), and JNK (1:1000), were purchased from Cell Signaling Technology (Danvers, MA, USA). Cleaved caspase-12 (1:1000) was purchased from Abcam (Cambridge, UK). After washing, the membranes were incubated with horseradish peroxidase-conjugated secondary antibodies (Cell Signaling Technology, Danvers, MA, USA) at dilution 1:2000 for 2 h at room temperature. The β-actin antibody was used as internal control. Finally, the membranes were incubated with chemiluminescence substrate and then exposed to X-ray film. The intensity of protein bands was quantified using the Image J program (National Institutes of Health, Bethesda, MD, USA) and normalized with β-actin. 

### 4.7. Measurement of Caspase-9 and Caspase-3 Activities by Assay Kit

The activities of caspase-9 and caspase-3 involved in the apoptotic cell death pathway were measured using commercial assay kits from Cayman Chemical (Ann Arbor, MI, USA) according to the manufacturer’s protocol. Briefly, the HMC3 cells were seeded in 6-well plates at a density of 1.5 × 10^5^ cells/well for 24 h. Then, cells were pre-treated with the extract for 24 h and then treated with glutamate for 30 min. After incubation, the cells were collected by centrifugation at 13,500 rpm at 4 °C for 10 min and then the supernatant was aspirated. Next, lysis buffer was added to each well. After 30 min, the plate was centrifuged again, and the supernatants transferred to a fresh well plate. Subsequently, the specific substrate of caspase-9 or caspase-3 was added to the wells. The caspase activity was determined by the fluorescence intensity (excitation at 485 nm and emission at 535 nm) by a CLARIOStar microplate reader (BMG LABTECH, Offenburg, Germany).

### 4.8. Determination of Antioxidant Activity by Assay Kits

The activities of antioxidants, namely SOD, CAT, and GPx, were measured using commercial assay kits from Cayman Chemical (Ann Arbor, MI, USA), following the manufacturer’s instructions. Briefly, the HMC3 cells were seeded in 6-well plates at a density of 1.5 × 10^5^ cells/well for 24 h. Then, cells were pre-treated with the extract for 24 h and then exposed to glutamate for 30 min. Next, cells were collected by centrifugation at 13,500 rpm at 4 °C for 10 min, the lysis buffer added, and the supernatant collected. The SOD, CAT, and GPx activities were evaluated after adding the specific reagents and measuring absorbance at 440 nm, 540 nm, and 340 nm, respectively, using a CLARIOStar microplate reader (BMG LABTECH, Offenburg, Germany).

### 4.9. Carotenoid Content

Carotenoids were analyzed using the high performance liquid chromatography (HPLC) technique. The procedure of carotenoid analysis was slightly modified from Praengam et al. [27]. Briefly, the AHE sample was performed using the saponification method with a modification from Ismail and Cheah [68]. Then, the sample was analyzed by HPLC using a Vydac 201TP54-C18 column (250 × 4.6 mm) and photodiode array detection. The mobile phase consists of acetonitrile: methanol: dichloromethane (80:11:9) with 0.1 g of ammonium acetate at a flow rate of 0.7 mL/min. The carotenoid contents were quantified by comparing retention times and spectral absorption with standard compounds (lutein, zeaxanthin, β-cryptoxanthin, α-carotene, and β-carotene) at a wavelength of 450 nm. Data are presented as µg/100 g fresh weight (FW).

### 4.10. Flavonoid Content 

The flavonoid contents were extracted and determined using the modified method from Dawilia et al. [69]. Briefly, the AHE sample was hydrolyzed with acid methanol to obtain an aglycone form. Then, the sample was boiled with 62.5% (*v*/*v*) methanol, t-butyl hydroquinone (0.5 g/L), and 6 N hydrochloric acid for 2 h. Next, the sample was placed on ice for 5 min and 0.1% (*w*/*v*) ascorbic acid solution added. Then, the sample was sonicated for 5 min and filtered through a 0.2 µm PTFE syringe filter. Samples were analyzed with HPLC (Agilent 1260 Series liquid chromatograph, USA) using a ZORBAX Eclipse XDB-C18 column (4.6 × 150 mm). The mobile phase consists of 0.1% trifluoroacetic acid (TFA) in water and 0.1% TFA in methanol. The flavonoid contents were quantified by comparing retention times and spectral absorption with standard compounds (myricetin, quercetin, kaempferol, luteolin, apigenin, naringenin, and hesperidin) The results are expressed as µg/g FW.

### 4.11. Statistical Analysis

Data were presented as mean ± standard deviation (SD). All results were performed in triplicate in at least three independent experiments. Differences between group means were determined using one-way ANOVA, followed by Tukey post hoc analysis at a significant level of *p* < 0.05. The data were analyzed by SPSS version 18.0 (SPSS Inc., Chicago, IL, USA).

## 5. Conclusions

**Figure 10:**
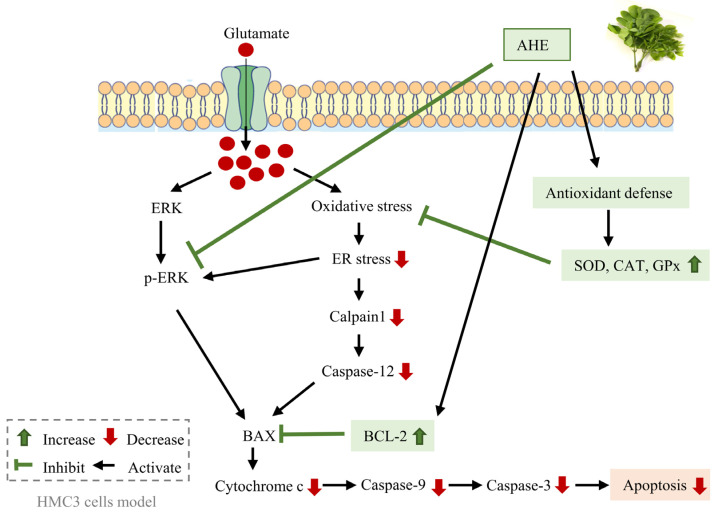
Schematic diagram of the proposed protective effect and mechanism of AHE against glutamate-induced neurotoxicity in HMC3 cells. Excessive glutamate neurotransmitters can induce ER stress and apoptosis signaling pathways in human microglial HMC3 cells. AHE reduces ERK phosphorylation and also suppresses apoptotic protein signaling. Moreover, AHE can up-regulate the anti-apoptotic protein and promote antioxidant enzymes, resulting in the inhibition of ER stress and apoptosis signaling pathways in HMC3 cells. (AHE, *A. lebbeck* leaf hexane extract).

This research reported, for the first time, about a beneficial indigenous edible leaf extract from *A. lebbeck* (L.) Benth on human microglial cells. Our study indicates that AHE inhibits glutamate-induced neurotoxicity through ER stress and apoptosis signaling pathways in human microglial HMC3 cells. AHE suppresses cell death by reducing ERK/MAPK phosphorylation, ER stress (calpain1 and cleaved caspase-12), and apoptosis signaling markers (Bax, caspase-9, and caspase-3). Meanwhile, AHE activates the anti-apoptotic Bcl-2 protein and the activities of antioxidant enzymes such as SOD, CAT, and GPx. The schematic diagram of the protective effect and mechanism of AHE in HMC3 cells is presented in Figure 10. However, further research is needed to investigate the pharmacodynamics and pharmacokinetic effects of *A. lebbeck* leaf extract on the neuronal and neuroglial systems in animal and human studies for proofing and providing a more comprehensive understanding of the neuroprotective effects of AHE. These findings support the potential of *A. lebbeck* leaf extract as a promising novel alternative plant for preventing neurodegenerative disorders. 

## Figures and Tables

**Table 1:** Carotenoid and flavonoid contents of AHE.

Carotenoids	Content (µg/100 g FW)
α-carotene	4706.57 ± 37.05
β-carotene	819.32 ± 24.85
lutein	4137.28 ± 162.20
zeaxanthin	ND
β-cryptoxanthin	ND
Total carotenoids	9663.17 ± 224.10
**Flavonoids**	**Content (µg/g FW)**
quercetin	989.42 ± 53.40
luteolin	254.67 ± 9.83
kaempferol	103.41 ± 9.05
myricetin	ND
apigenin	ND
naringenin	ND
hesperidin	ND
Total flavonoids	1347.50 ± 72.28

Values are expressed as mean ± SD (*n* = 3); Not detected (ND); Fresh weight (FW); *A. lebbeck* leaf hexane extract (AHE).

## Data Availability

Data are contained within the article.

## Supplementary Materials

The following supporting information can be downloaded at: https://www.mdpi.com/article/10.3390/ph16070989/s1, Figure S1: Original photographs for the full-length blots at three independent experiments of each protein marker of Figure 3A; Figure S2: Original photographs for the full length blots at three independent experiments of each protein marker of Figure 4A; Figure S3: Original photographs for the full length blots at three independent experiments of each protein marker of Figure 5A.
