# Supplementary material for: Neuroprotective Effects of Albizia lebbeck (L.) Benth. Leaf Extract against Glutamate-Induced Endoplasmic Reticulum Stress and Apoptosis in Human Microglial Cells
Source: Pharmaceuticals (Basel). 2023 Jul 10;16(7):989. doi: 10.3390/ph16070989 (PMC10384906; doi:10.3390/ph16070989)

Original Images for Blots/Gels

Supplementary Figure S1. Original photographs for the full-length blots at three independent experiments of each protein marker of Figure 3A.

<u>Figure 3A</u>

Calpain1

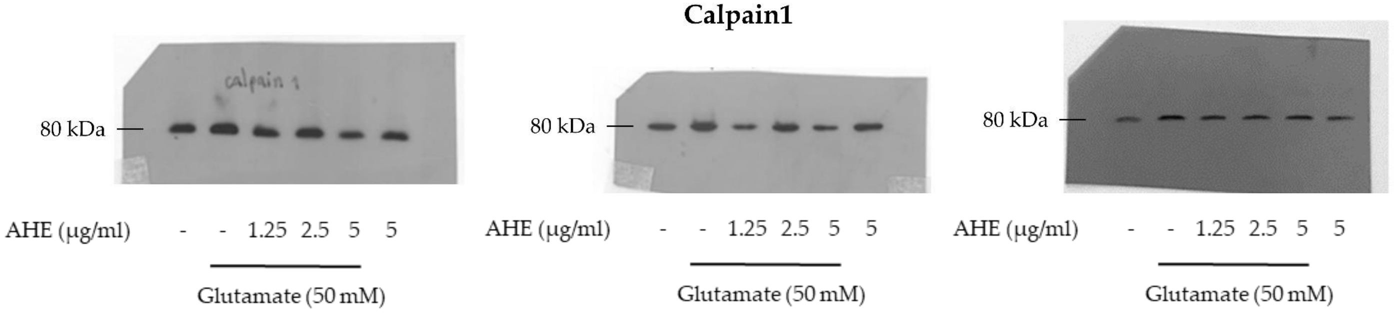

β-actin

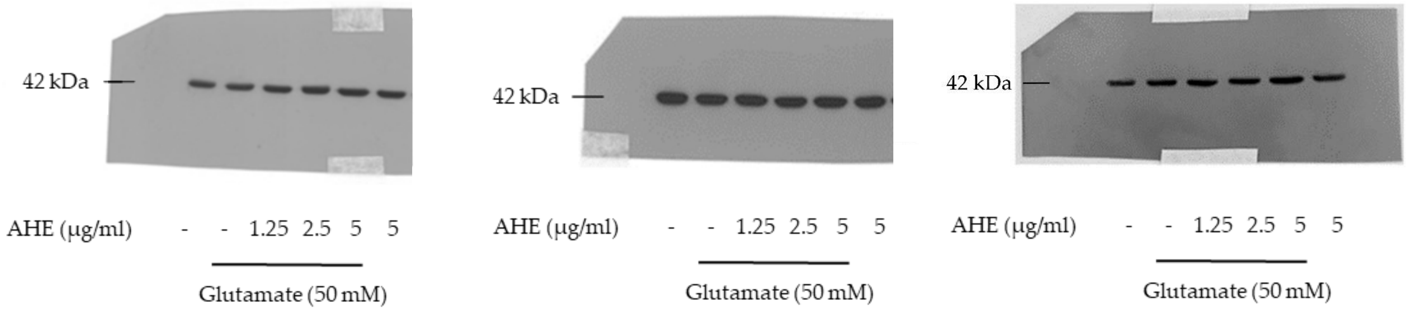

Cleaved caspase-12

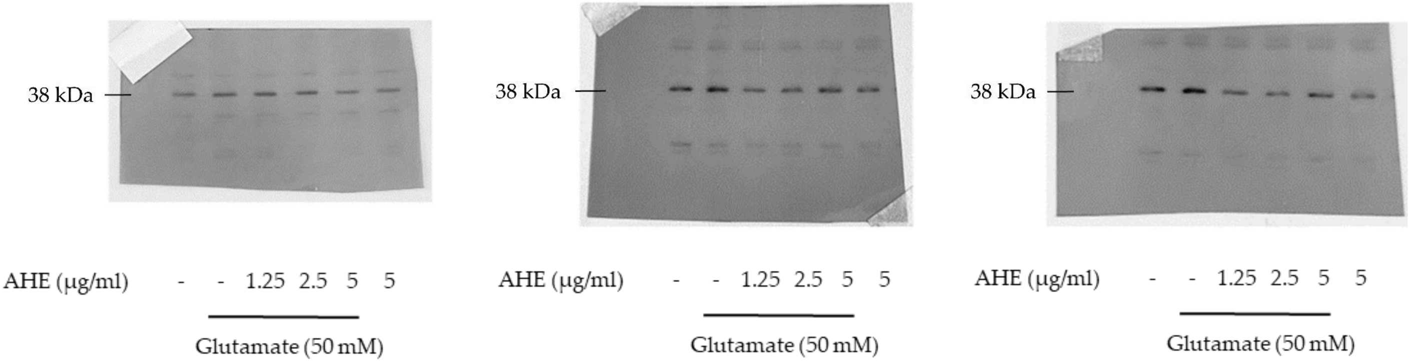

β-actin

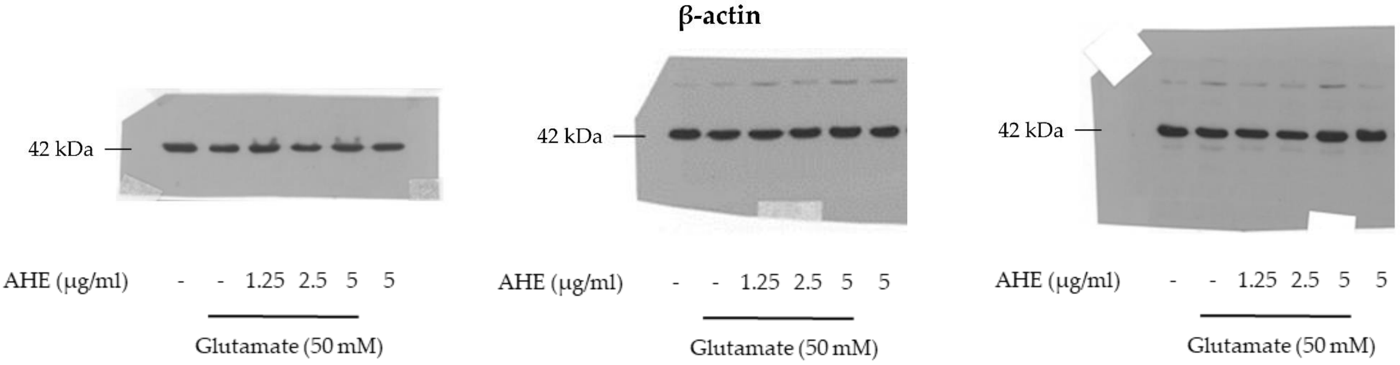

CHOP

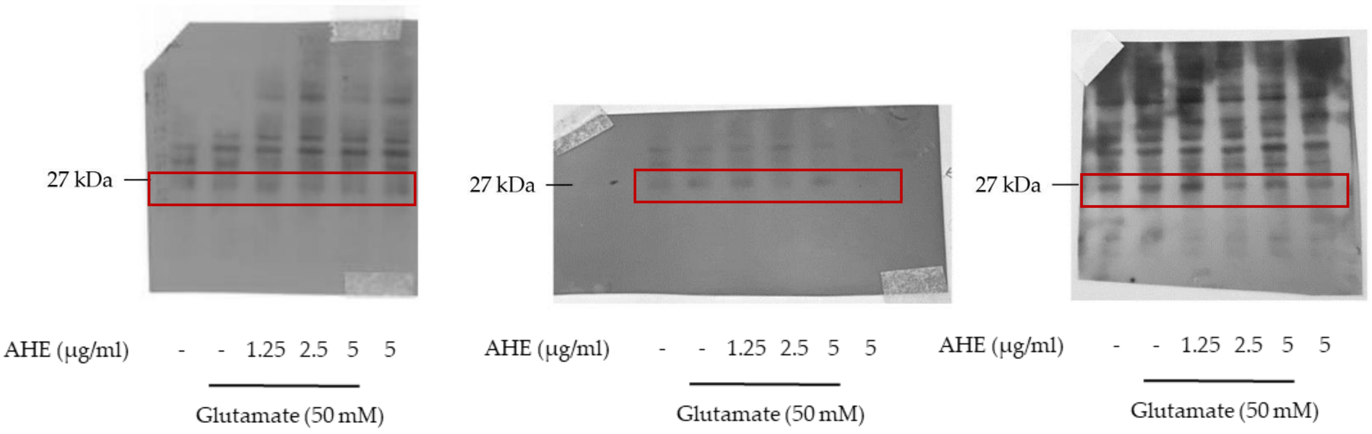
27 kDa
27 kDa
27 kDa
AHE (µg/ml) - - 1.25 2.5 5 5
Glutamate (50 mM)
AHE (µg/ml) - - 1.25 2.5 5 5
Glutamate (50 mM)
AHE (µg/ml) - - 1.25 2.5 5 5
Glutamate (50 mM)

β-actin

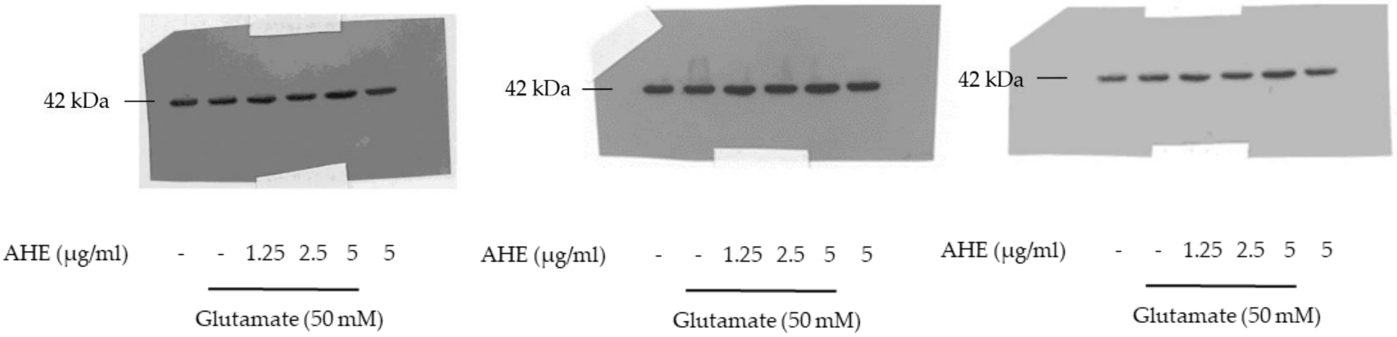
42 kDa
42 kDa
42 kDa
AHE (µg/ml) - - 1.25 2.5 5 5
Glutamate (50 mM)
AHE (µg/ml) - - 1.25 2.5 5 5
Glutamate (50 mM)
AHE (µg/ml) - - 1.25 2.5 5 5
Glutamate (50 mM)

Supplementary Figure S2. Original photographs for the full-length blots at three independent experiments of each protein marker of Figure 4A.

<u>Figure 4A</u>

p-ERK

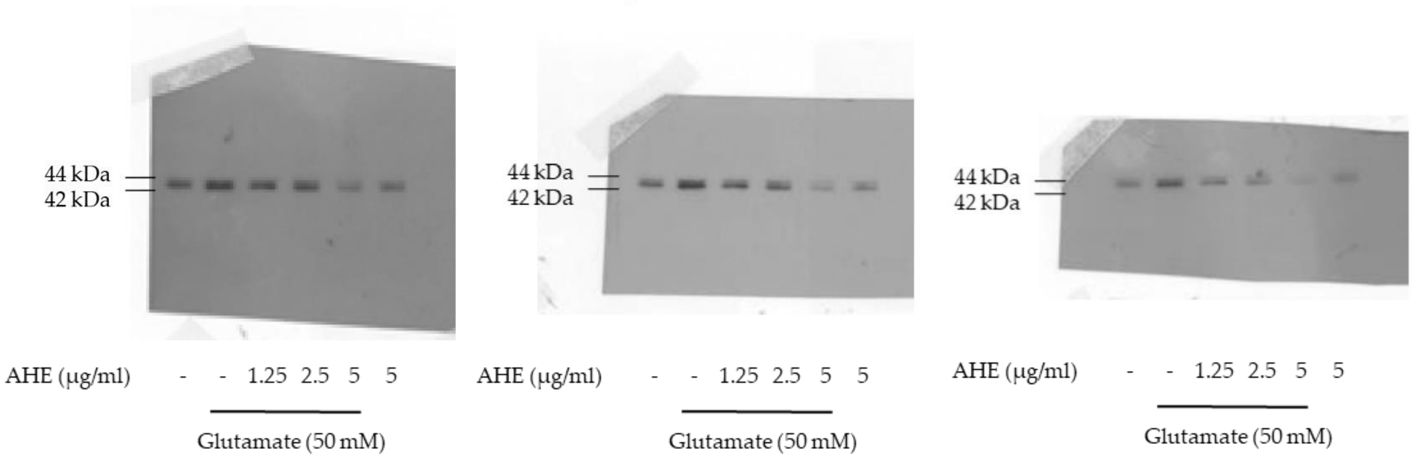

ERK

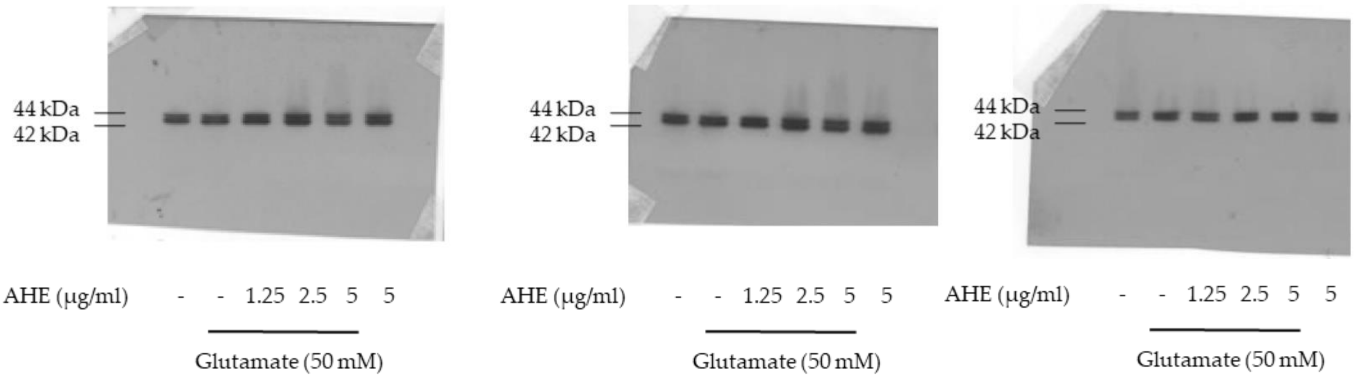

β-actin

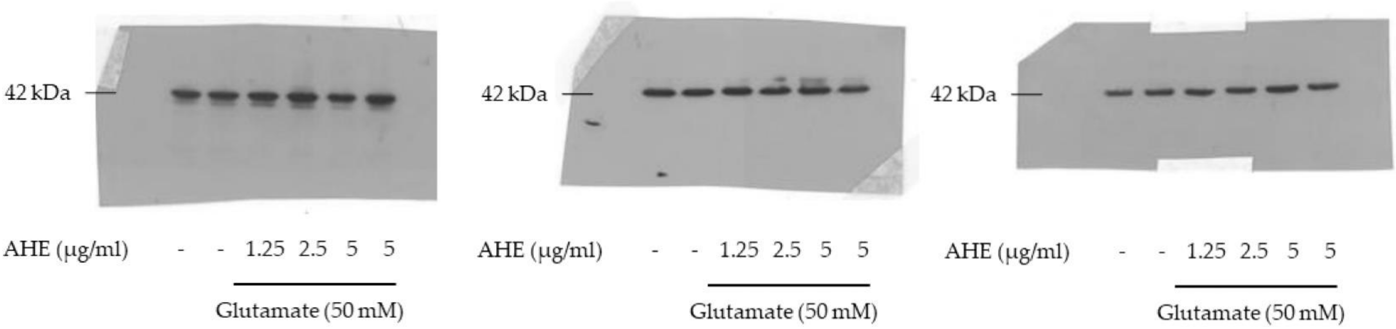

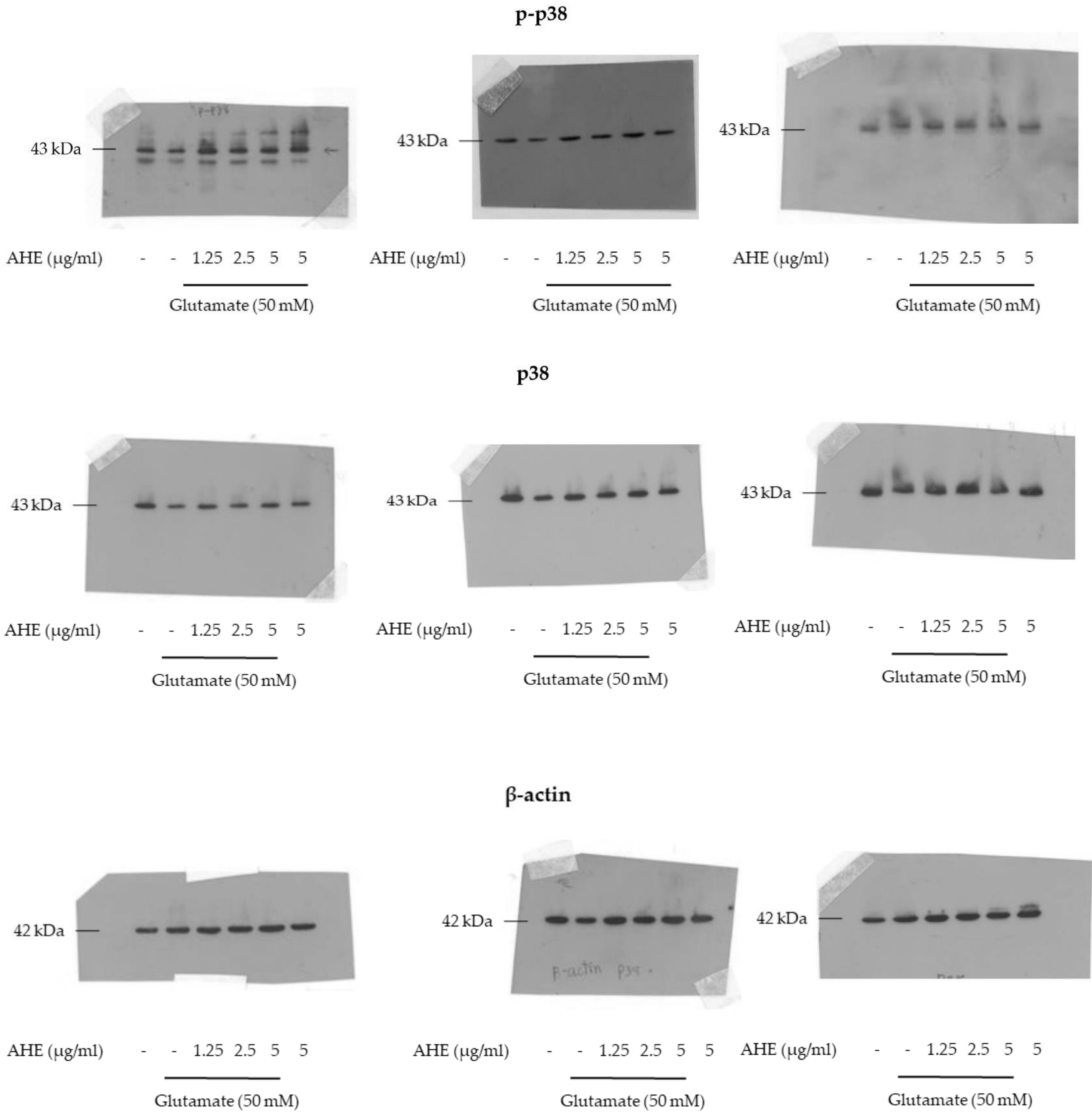

p-p38
43 kDa
AHE (μg/ml) - - 1.25 2.5 5 5
Glutamate (50 mM)
p38
43 kDa
AHE (μg/ml) - - 1.25 2.5 5 5
Glutamate (50 mM)
β-actin
42 kDa
AHE (μg/ml) - - 1.25 2.5 5 5
Glutamate (50 mM)

P-JNK

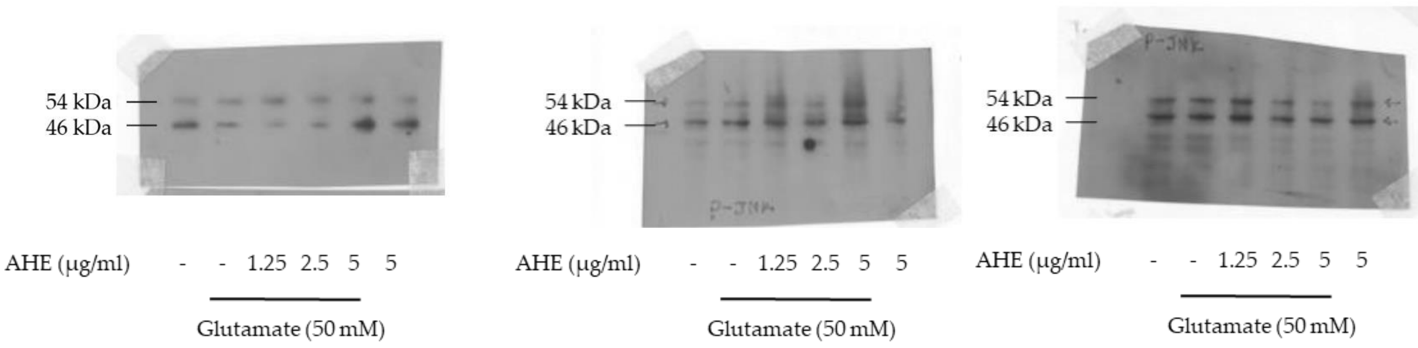

JNK

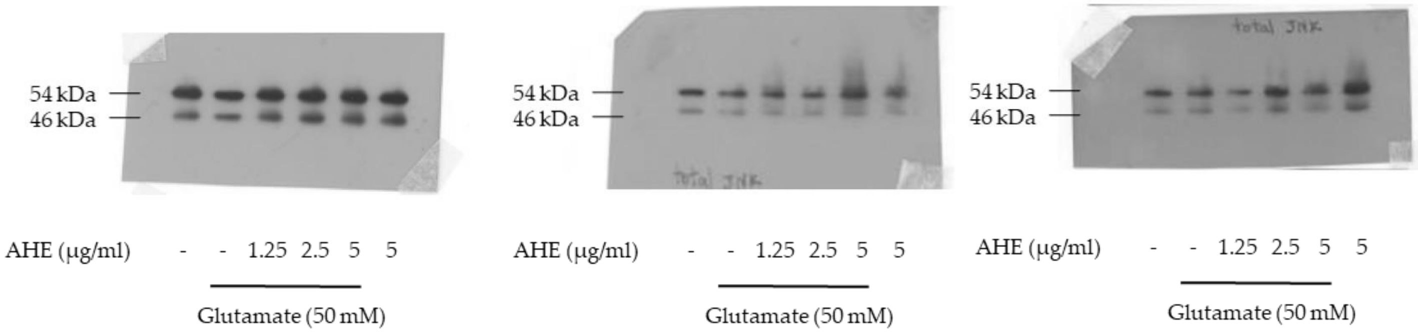

β-actin

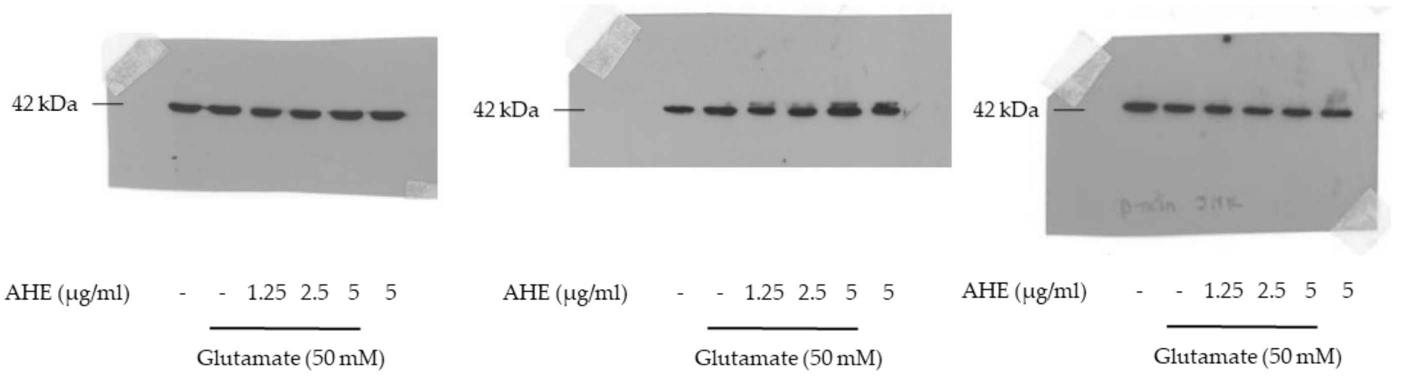

Supplementary Figure S3. Original photographs for the full-length blots at three independent experiments of each protein marker of Figure 5A.

<u>Figure 5A</u>

BAX

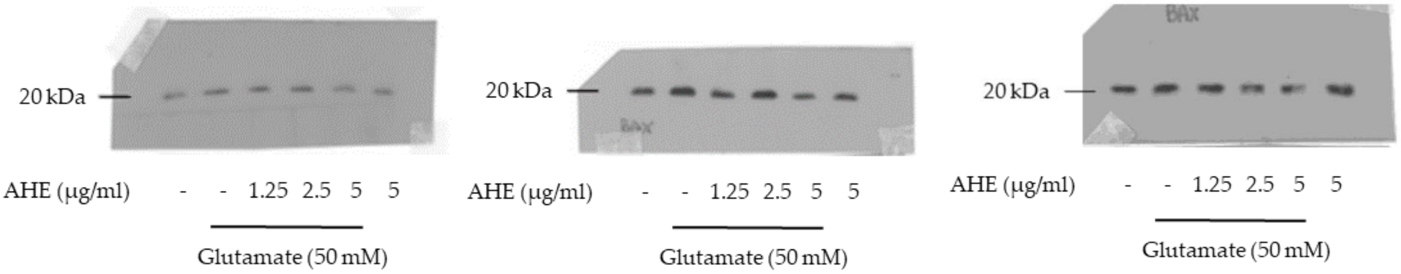

β-actin

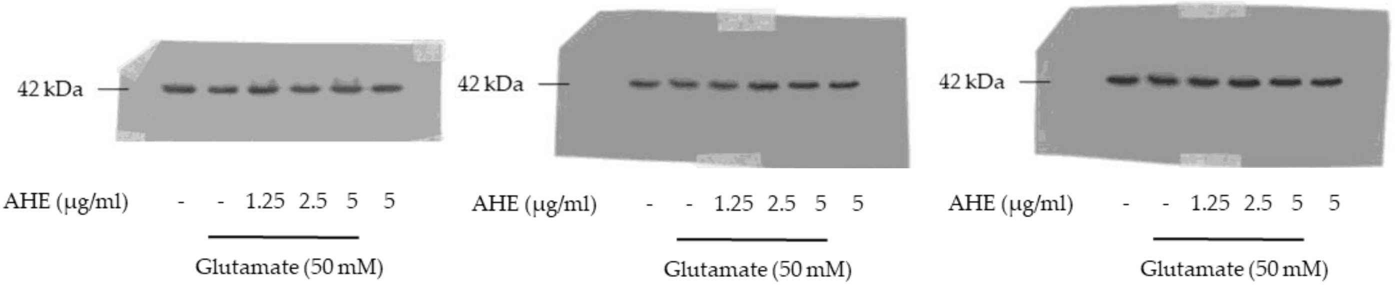

Bcl-2

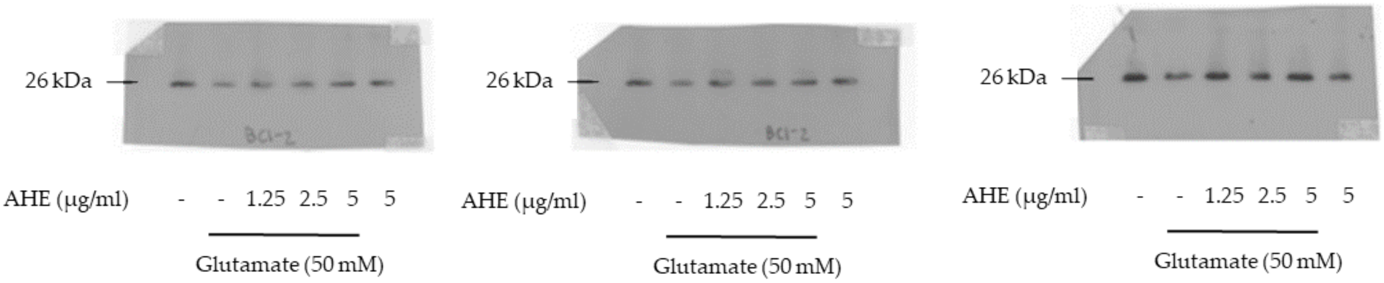

β-actin

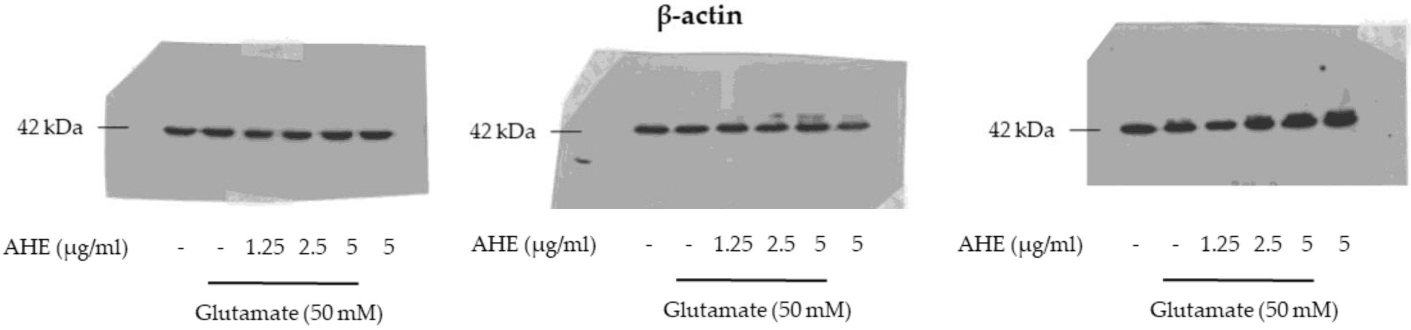

Cytochrome c

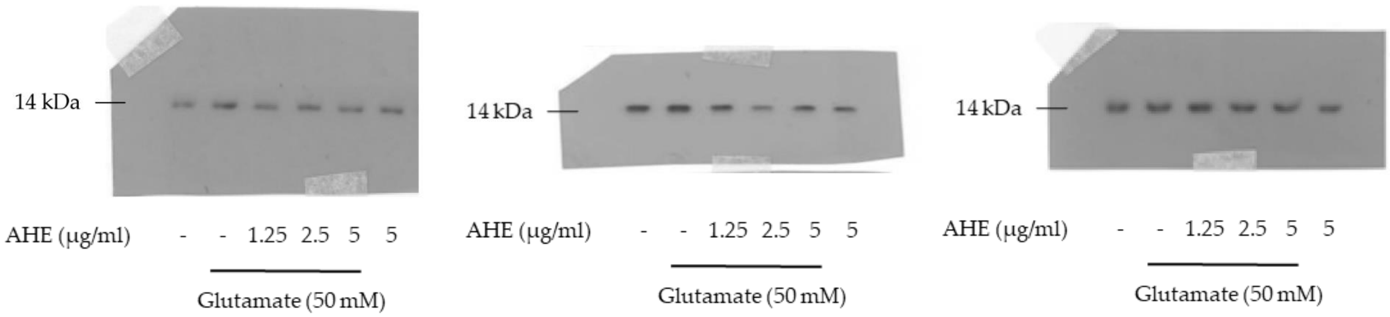

β-actin

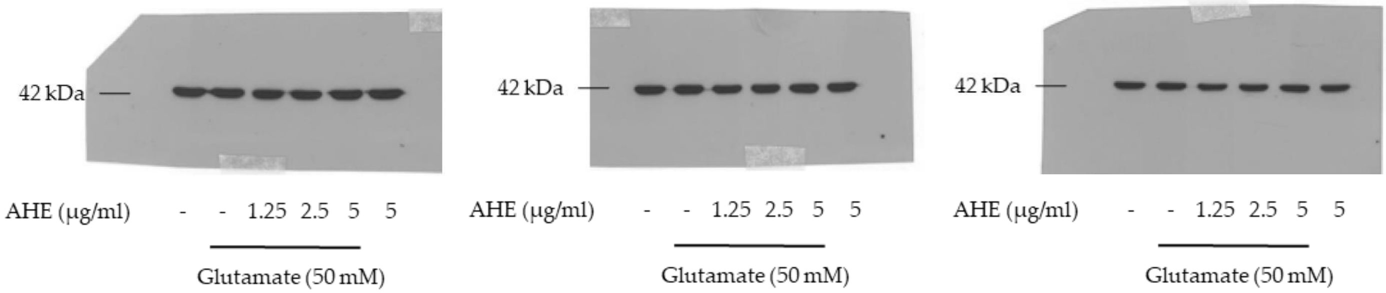

Cleaved caspase-9

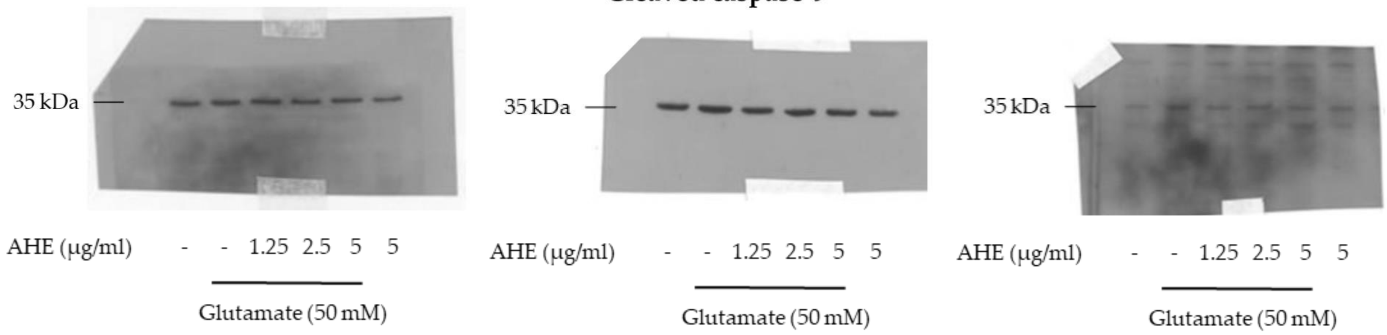

β-actin

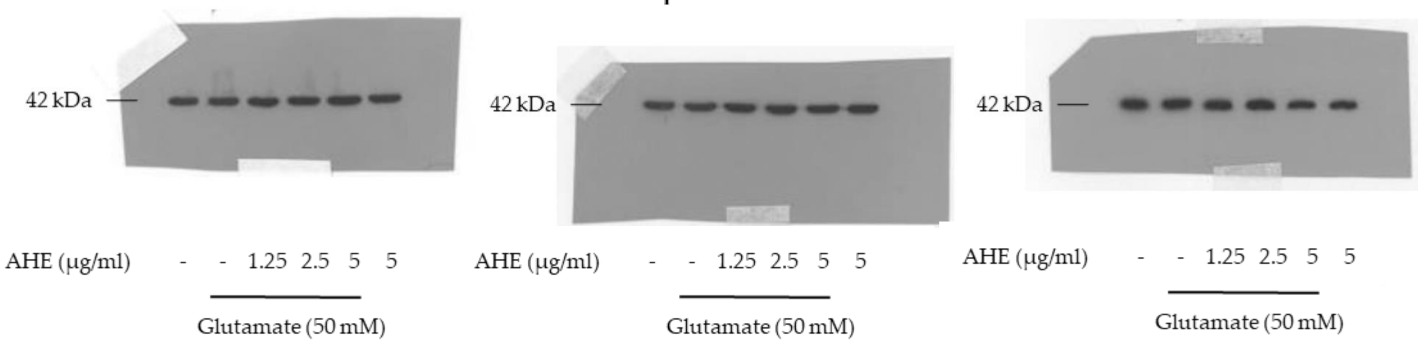

Supplement: Supplementary file 1 [file pharmaceuticals-16-00989-s001.zip › pharmaceuticals-2483756-supplementary.pdf]
